# Relationship Between Glycosylated Hemoglobin and Short-Term Mortality of Spontaneous Intracerebral Hemorrhage

**DOI:** 10.3389/fneur.2021.648907

**Published:** 2021-04-16

**Authors:** Ping Lu, Lingyun Cui, Yu Wang, Kaijiang Kang, Hongqiu Gu, Zixiao Li, Liping Liu, Yilong Wang, Xingquan Zhao

**Affiliations:** ^1^Department of Neurology, Beijing Tiantan Hospital, Capital Medical University, Beijing, China; ^2^China National Clinical Research Center for Neurological Diseases, Beijing, China; ^3^Research Unit of Artificial Intelligence in Cerebrovascular Disease, Chinese Academy of Medical Sciences, Beijing, China

**Keywords:** HbA1c, glucose, diabetes, intracerebral hemorrhage, mortality

## Abstract

**Background:** The relationship between glycosylated hemoglobin (HbA1c) and prognosis of spontaneous intracerebral hemorrhage (SICH) patients has not been fully elucidated. This study aimed to reveal the relationship between HbA1c levels and short-term mortality after patient admission with SICH.

**Methods:** It was a large-scale, multicenter, cross-sectional study. From August 1, 2015, to July 31, 2019, a total of 41910 SICH patients were included in the study from the Chinese Stroke Center Alliance (CSCA) program. Finally, we comprehensively analyzed the data from 21,116 patients with SICH. HbA1c was categorized into four groups by quartile. Univariate and multivariate logistic regression analyses were used to assess the association between HbA1c levels and short-term mortality in SICH patients.

**Results:** The average age of the 21,116 patients was 62.8 ± 13.2 years; 13,052 (61.8%) of them were male, and 507 (2.4%) of them died. Compared to the higher three quartiles of HbA1c, the lowest quartile (≤5.10%) had higher short-term mortality. In subgroup analysis with or without diabetes mellitus (DM) patients, the mortality of the Q3 group at 5.60–6.10% was significantly lower than that of the Q1 group at ≤5.10%. After adjustment for potential influencing factors, the ROC curve of HbA1c can better predict the short-term mortality of patients with SICH (AUC = 0.6286 *P* < 0.001).

**Conclusions:** Therefore, we concluded that low or extremely low HbA1c levels (≤5.10%) after stroke were associated with higher short-term mortality in SICH patients, with or without DM.

## Introduction

Spontaneous intracerebral hemorrhage (SICH) accounts for 20–30% of all strokes. As a disabling type of stroke with poor prognosis, SICH contributes to an increase in the global burden of the disease (1, 2). The 30-day mortality rate of ICH was 35–52% (3). Half of the deaths occurred in the acute phase, especially in the first 2 days (4, 5).

Data from several studies suggested that hyperglycemia is associated with severe neurological impairment and poor prognosis in SICH patients (6–14). It is recommended that blood glucose levels should be measured and closely monitored to avoid hyperglycemia (15). However, in patients with SICH (16–18), subarachnoid hemorrhage (19), and ischemic stroke (20), early intensive insulin hypoglycemic therapy did not improve functional prognosis. Recent studies demonstrated that hyperglycemia is only the result of severe nervous system damage, which may be caused by a stress response, mainly adrenergic stress, and relative insulin deficiency (21, 22). Thus, it suggests that blood glucose level measured after the onset of SICH is not an ideal prognostic indicator for stroke patients. In contrast, glycosylated hemoglobin (HbA1c) is a measure of average blood glucose across 2–3 months before stroke. HbA1c possesses a high stability than random blood glucose after stroke, without the need to be measured or compared in a specific time (23, 24). Therefore, HbA1c could be considered as a potential biomarker for the prognosis of SICH. Some studies revealed that HbA1c is a better predictor of adverse outcomes in patients with SICH (25–27). However, a study showed that HbA1c is not associated with clinical outcome in patients with SICH (28). The relationship between HbA1c and the prognosis of SICH patients is not yet fully elucidated.

Therefore, our study aimed to investigate the relationship between HbA1c levels and short-term mortality in SICH patients.

## Methods

### Study Population

From August 1, 2015, to July 31, 2019, a total of 1,006,798 patients diagnosed with cerebral hemorrhage, subarachnoid hemorrhage, acute ischemic stroke, or transient ischemic attack were included in the Chinese Stroke Center Alliance (CSCA) program. The patients were over 18 years old within 7 days of symptom onset. In the CSCA program, there was no follow-up after discharge, and only in-hospital information was recorded. We comprehensively analyzed the data from SICH patients enrolled in the CSCA. We included all the SICH patients. Spontaneous intracerebral hemorrhage refers to intracerebral hemorrhage caused by spontaneous rupture of the cerebral artery, vein, and capillary, excluding traumatic cerebral hemorrhage. We excluded patients with (1) history of previous stroke events; (2) history of abnormal liver function and renal function; (3) bleeding history or tendency; (4) lack data of death; and (5) lack data of HbA1c.

The study was conducted in accordance with guidelines from the Helsinki Declaration. All participating hospitals of CSCA had the right to collect data without informed consent of patients or a waiver of authorization and exemption from their institutional review board.

### Clinical Information

During the hospital admission of the selected patients for this study, the following information was collected: demographic information, medical history [atrial fibrillation, coronary heart disease, hypertension, diabetes mellitus, dyslipidemia, peripheral vascular disease, chronic obstructive pulmonary disease (COPD), drinking, smoking, body mass index (BMI), medication history (hypotension, hypoglycemia, anticoagulation, antiplatelet agent), systolic blood pressure (SBP), and diastolic blood pressure (DBP)]. A history of diabetes is defined as a patient who was definitely diagnosed with diabetes before admission.

### Baseline Neurological Assessment

The Glasgow Coma Scale (GCS) and National Institutes of Health Stroke Scale (NIHSS) were used to assess neurological deficit at admission. The GCS score was divided into mild coma (13–15) or moderate to severe coma (≤12). The NIHSS score was divided into mild to moderate disability (<16) or severe disability (≥16).

### Laboratory Examinations

Venous blood was collected in a vacuum EDTA collection tube, and plasma was separated. HbA1c was assessed within 7 days after admission. Patients with SICH were divided into four groups according to the quartile of HbA1c: Q1, HbA1c ≤5.10%; Q2, HbA1c 5.10–5.60%; Q3, HbA1c 5.60–6.10%; and Q4, HbA1c ≥6.10%. The quartile was based on the data available in the study. There were significant differences in HbA1c levels among different quartile groups. Compared with the previously published data, the quartile level of HbA1c determined in this study is generally low.

Other laboratory tests, including FBG, bun, creatinine, low-density lipoprotein cholesterol (LDL-C), serum homocysteine (HCY), and international normalized ratio (INR) were also collected.

### Clinical Outcomes

We analyzed the relationship between HbA1c levels and adverse outcome in patients with SICH. Adverse outcomes were defined as death during hospitalization.

### Statistical Analysis

Continuous variables were expressed as mean ± standard deviation; categorical variables were presented as count (percentage). The median and quartile range expressed ordinal variables. Group differences were analyzed by the independent sample *t*-test or the Mann–Whitney *U*-test for continuous variables and by the chi-squared test for categorical variables. Logistic regression was used to analyze the relationship between different glycosylated hemoglobin levels and short-term mortality. The receiver operating characteristic (ROC) curve was used to evaluate the prognostic value of HbA1c. Sensitivity analysis was used to estimate the effects of potential unmeasured or uncontrolled confounding variables. First, we coded patients who lacked the NIHSS score or GCS score. Then, we analyzed patients with SICH who had NIHSS and GCS scores to determine if the results were similar. Odds ratios (ORs) and 95% confidence interval (95% CI) were expressed for the results and probability values. A two-sided value of *P* < 0.05 was considered statistically significant. All statistical analyses were performed using SAS software, version 9.4 (SAS Institute, Cary, NC, USA).

## Results

### Baseline Characteristics

**Figure 1:**
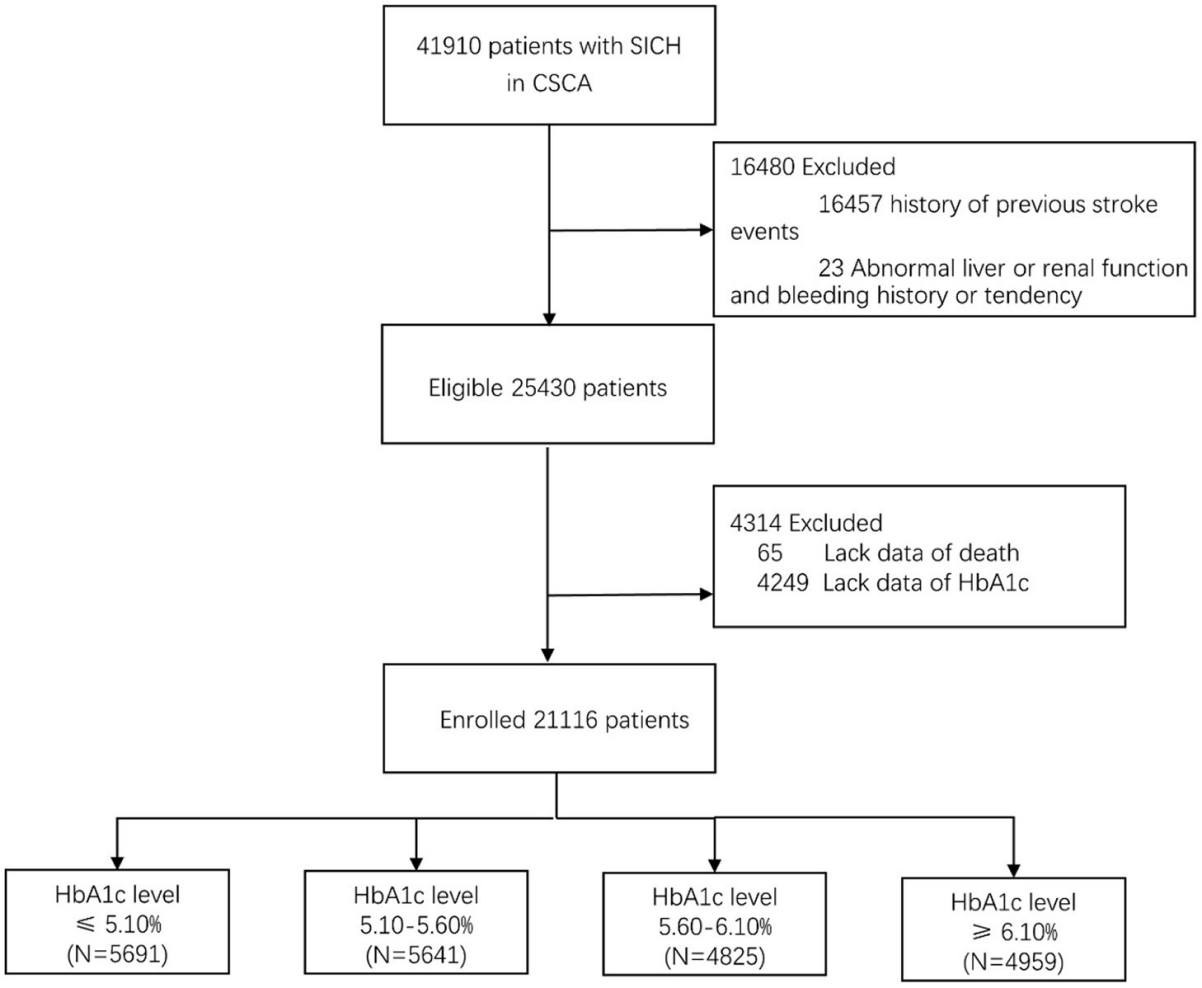
Study population flowchart.

From August 1, 2015, to July 31, 2019, a total of 41,910 SICH patients were included in the study from the CSCA program. We excluded patients with history of previous stroke events, abnormal liver function, renal function, and bleeding history or tendency. A total of 25,430 patients with SICH were enrolled, and 21,116 (83.0%) patients of them had valid data of HbA1c and death while the data for others were missing (Figure 1). The average age of the patients having valid data was 62.8 ± 13.2 years; 13,087 (61.8%) of them were male, and 507 (2.4%) of them died. Age, atrial fibrillation, myocardial infarction, hypertension, diabetes, and hyperlipidemia in patients with HbA1c stratification of ≥6.10% were larger than in patients with HbA1c stratification of ≤5.10%. With regard to sex, the highest quartile group presented relatively few males. Patients with higher HbA1c levels were less likely to drink or smoke and more likely to be obese. Table 1 shows the baseline characteristics of 21,116 patients with different HbA1c levels. Due to the missing HbA1c in more than 15%, a comparison of baseline characteristics between valid and missing HbA1cs was conducted. This comparison revealed significant differences in GCS and NIHSS scores between valid and missing HbA1c. We observed that GCS was relatively low and NIHSS was relatively high in the group lacking HbA1c data (Table 2).

**Table 1:** Baseline characteristics of SICH patients according to HbA1c quartiles.

**Variables**	**Q1: ≤5.10% (*N* = 5,691)**	**Q2: 5.10–5.60% (*N* = 5,641)**	**Q3: 5.60–6.10% (*N* = 4,825)**	**Q4: ≥6.10% (*N* = 4,959)**	***P*-value**
Age, years	61.1 ± 13.7	62.5 ± 13.4	63.8 ± 12.8	64.0 ± 12.4	<0.001
Male	3,621 (63.6)	3,566 (63.2)	2,951 (61.2)	2,914 (58.8)	<0.001
BMI	23.7 ± 5.0	23.7 ± 3.8	23.8 ± 3.7	24.3 ± 5.2	<0.001
Systolic BP (mmHg)	163.4 ± 29.4	164.0 ± 27.9	164.0 ± 28.2	164.5 ± 28.8	0.102
Diastolic BP (mmHg)	95.4 ± 17.3	95.0 ± 17.1	94.2 ± 16.8	94.0 ± 17.1	<0.001
Atrial fibrillation	70 (1.2)	87 (1.5)	102 (2.1)	118 (2.4)	<0.001
Myocardial infarction	41 (0.7)	40 (0.7)	49 (1.0)	70 (1.4)	<0.001
Hypertension	3,567 (62.7)	3,620 (64.2)	3,145 (65.2)	3,534 (71.3)	<0.001
Diabetes mellitus	150 (2.6)	114 (2.0)	201 (4.2)	1,498 (30.2)	<0.001
Dyslipidemia	151 (2.7)	125 (2.2)	129 (2.7)	260 (5.2)	<0.001
Peripheral vascular disorder	25 (0.4)	29 (0.5)	25 (0.5)	52 (1.0)	0.001
COPD	72 (1.3)	87 (1.5)	77 (1.6)	89 (1.8)	0.168
Antiplatelet	180 (3.2)	188 (3.3)	186 (3.9)	271 (5.5)	<0.001
Anticoagulation	76 (1.3)	69 (1.2)	53 (1.1)	81 (1.6)	0.009
Antihypertensive	2,098 (36.9)	2,208 (39.1)	1,982 (41.1)	2,447 (49.3)	<0.001
Diabetic medication	101 (1.8)	68 (1.2)	132 (2.7)	1,099 (22.2)	<0.001
Current smoking	1,263 (22.2)	1,304 (23.1)	975 (20.2)	884 (17.8)	<0.001
drinking	1,528 (26.8)	1,518 (26.9)	1,176 (24.4)	1,183 (23.9)	<0.001
NIHSS	6.0 (2.0-12.0)	6.0 (2.0-12.0)	5.0 (2.0-12.0)	6.0 (2.0-14.0)	<0.001
GCS	13.0 (7.0-15.0)	14.0 (8.0-15.0)	14.0 (8.0-15.0)	13.0 (7.0-15.0)	<0.001
Hypoglycemic treatment	114 (2.0)	87 (1.5)	158 (3.3)	1,585 (32.0)	<0.001

*Continuous variables were expressed as mean ± SD; categories variables were expressed as n (%). BMI, body mass index; COPD, chronic obstructive pulmonary disease; NIHSS, National Institutes of Health Stroke Scale; GCS, Glasgow Coma Scale. Proportion of NIHSS missing (39.8, 38.0, 39.3, and 40.4%). Proportion of GCS missing (47, 46.4, 46, and 48.5%)*.

**Table 2:** Baseline characteristics between valid and missing HbA1c.

**Variables**	**Missing (*N* = 4,244)**	**Valid (*N* = 21,116)**	***P*-value**
Age, years	61.7 ± 13.6	62.8 ± 13.2	<0.001
Male	2,619 (61.7)	13,052 (61.8)	0.902
BMI	23.8 ± 4.8	23.9 ± 4.5	0.638
Systolic BP (mmHg)	163.3 ± 30.7	163.9 ± 28.6	0.021
Diastolic BP (mmHg)	94.9 ± 17.5	94.7 ± 17.1	0.381
Atrial fibrillation	67 (1.6)	377 (1.8)	0.359
Myocardial infarction	47 (1.1)	200 (0.9)	0.608
Hypertension	2,607 (61.4)	13,866 (65.7)	<0.001
Diabetes mellitus	205 (4.8)	1,963 (9.3)	<0.001
Dyslipidemia	90 (2.1)	665 (3.1)	0.002
Peripheral vascular disorder	17 (0.4)	131 (0.6)	0.125
COPD	61 (1.4)	325 (1.5)	0.621
Antiplatelet	104 (2.5)	825 (3.9)	<0.001
Anticoagulation	50 (1.2)	279 (1.3)	0.281
Antihypertensive	1,536 (36.2)	8,735 (41.4)	<0.001
Diabetic medication	148 (3.5)	1,400 (6.6)	<0.001
Current smoking	952 (22.4)	4,426 (21.0)	<0.001
Drinking	1,044 (24.6)	5,405 (25.6)	0.394
NIHSS	7.0 (3.0-14.0)	6.0 (2.0-12.0)	<0.001
GCS	13.0 (7.0-15.0)	13.0 (8.0-15.0)	0.002
Hypoglycemic treatment	162 (3.8)	1,944 (9.2)	<0.001

*Continuous variables were expressed as mean ± SD; categories variables were expressed as n (%). BMI, body mass index; COPD, chronic obstructive pulmonary disease; NIHSS, National Institutes of Health Stroke Scale; GCS, Glasgow Coma Scale; NIHSS and GCS in valid data of HbA1c were partially missing (39.3 and 47.0%)*.

### Logistic Regression Analysis

Among the patients with different HbA1c levels, the univariate logistic regression analysis showed that in the HbA1c stratification of 5.10–5.60% and 5.60–6.10% of patients, the short-term mortality rate after admission was drastically lower than that of patients in the ≤5.10% group (OR respectively 0.74 95% CI *P* = 0.014 and 0.66 95% CI *P* = 0.002). However, no significant association was found in the ≥6.10% group compared with the ≤5.10% group (OR: 1.15 95% CI, *P* = 0.244). The results were consistent after the imbalance factors were adjusted (Table 3).

**Table 3:** Association between HbA1c quartiles with mortality after intracerebral hemorrhage.

**HbA1c**	***N***	**Death, *n* (%)**	**Univariate analysis**		Multivariate analysis*	
			***P-*value**	**OR (95%CI)**	***P-*value**	**aOR (95%CI)**
≤5.10%	5,691	154 (2.71)	-	-		
5.10–5.60%	5,641	113 (2.00)	0.014	0.74 (0.58–0.94)	0.006	0.70 (0.55–0.90)
5.60–6.10%	4,825	87 (1.80)	0.002	0.66 (0.51–0.86)	0.001	0.63 (0.48–0.83)
≥6.10%	4,959	153 (3.09)	0.244	1.15 (0.91–1.44)	0.925	0.99 (0.77–1.27)

The multivariate model* was adjusted for age, sex, BMI, diastolic blood pressure, atrial fibrillation, myocardial infarction, hypertension history, diabetes history, lipid metabolism disorder, peripheral vascular disease, antiplatelet drugs, antihypertensive drugs, hypoglycemic drugs, smoking history, drinking history, and hypoglycemic therapy after the onset of the disease. GCS and NIHSS are not included.

### Subgroup Analysis

A stratified analysis of the association between HbA1c levels and short-term mortality after admission in patients of SICH with or without DM was performed. In univariate analysis of the patients with DM, a significant difference of the short-term mortality was found in the 5.60–6.10% group compared with the ≤5.10% group (*P* > 0.05). However, there was no significant correlation between HbA1c 5.10–5.60% and ≥6.10% groups compared with the ≤5.10% (*P* > 0.05) group. In the patients without DM, there was a significant correlation between the HbA1c 5.10–5.60% and 5.60–6.10% groups compared with ≤5.10% (*P* < 0.05). However, compared with the ≤5.10% group, no significant association was found in the ≥6.10% group (*P* > 0.05). The conclusions were consistent after adjusting the imbalance factors (Table 4).

**Table 4:** Stratified analysis of association between HbA1c levels and short-term mortality after admission in patients of SICH with or without DM.

**HbA1c**	***N***	**Death, *n* (%)**	**DM**				***N***	**Death, *n* (%)**	**Non-DM**			
			**Univariate analysis**		Multivariate analysis*				**Univariate analysis**		Multivariate analysis*	
			***P*-value**	**OR (95%CI)**	***P-*value**	**aOR (95%CI)**			***P-*value**	**OR (95%CI)**	***P-*value**	**aOR (95%CI)**
≤5.10%	150	9 (6.00)	–	–	–	–	5,541	145 (2.62)	–	–	–	–
5.10–5.60%	114	2 (1.75)	0.108	0.28 (0.06–1.32)	0.112	0.28 (0.06–1.35)	5,527	111 (2.01)	0.034	0.76 (0.59–0.98)	0.017	0.73 (0.57–0.95)
5.60–6.10%	201	2 (1,00)	0.019	0.16 (0.03–0.74)	0.012	0.13 (0.03–0.64)	4,624	85 (1.84)	0.009	0.70 (0.53–0.91)	0.005	0.68 (0.51–0.89)
≥6.10%	1,498	53 (3.54)	0.136	0.58 (0.28–1.19)	0.074	0.50 (0.24–1.07)	3,461	100 (2.89)	0.438	1.11 (0.86–1.43)	0.812	1.03 (0.79–1.35)

The multivariate model* was adjusted for age, male, BMI, diastolic blood pressure, atrial fibrillation, myocardial infarction, hypertension history, diabetes history, lipid metabolism disorder, peripheral vascular disease, antiplatelet drugs, antihypertensive drugs, hypoglycemic drugs, smoking history, drinking history, and hypoglycemic therapy after the onset of the disease. GCS and NIHSS are not included.

### The ROC Curve

The AUC was 0.5596 (*P* < 0.001). In addition, the ROC curve of HbA1c combined with age, male, BMI, diastolic blood pressure, atrial fibrillation, myocardial infarction, hypertension history, diabetes history, lipid metabolism disorder, peripheral vascular disease, antiplatelet drugs, antihypertensive drugs, hypoglycemic drugs, smoking history, drinking history, NIHSS score, GCS, and hypoglycemic therapy after the onset of the disease can better predict the short-term mortality of patients with SICH (AUC = 0.6286 *P* < 0.001).

## Discussion

In this large, multicenter, cross-sectional study, we concluded that low or extremely low HbA1c after the stroke is associated with higher short-term mortality after SICH, regardless of whether patients have DM or not.

In accordance with the present results, previous studies have shown that very low HbA1c (<5.0 or <4.0%) is an independent risk factor for all-cause mortality (29, 30). The mechanism between low or very low HbA1c and higher short-term mortality after SICH has not been fully elucidated, which will be speculated as follows. Accounting for 20% of the total body energy consumption, brain energy consumption is mainly through sugar to provide energy. In light of the limited energy storage material, it is easy to be affected by the decrease of substrate supply (31). The increase of blood glucose is regulated by glucagon, adrenaline, and other hormones, of which glucagon and epinephrine mainly act on the liver, promoting the decomposition of liver glycogen into the blood and increasing the gluconeogenesis. Low HbA1c represents that the blood glucose level before stroke is at a low level for a long time, which transfers the blood glucose threshold of adrenaline to a low plasma glucose level, reducing the response level of elevated blood glucose and slowing the response of glucagon (32). It may lead to “brain energy crisis” (18, 33–35) when SICH occurs in this group of people by virtue of the low blood glucose threshold of adrenaline and the slow response of glucagon, hereby resulting in the increase of brain anaerobic metabolism and increasing the short-term mortality. It is found that the metabolic demand is the highest on the third day of intracranial injury (36, 37). If the patient has liver fibrosis or cirrhosis at the same time, the ability of the liver to regulate glucose will be affected. In this case, intrahepatic gluconeogenesis and glycogen decomposition will be further reduced, which will aggravate intracranial glucose deficiency. At the same time, the levels of procoagulant factor and anticoagulant factor were decreased and vitamin K was deficient in patients with abnormal liver function, which may generate larger hematoma volume and higher risk of rebleeding for patients with SICH.

Low or extremely low HbA1c after stroke may be related to the following aspects. To begin with, low or extremely low HbA1c may be associated with potential liver disease (38). Although we excluded patients with a history of liver dysfunction that had been diagnosed before admission, potential liver dysfunction or liver vulnerability may exist in the included population. Additionally, a very low HbA1c level may indicate the long-term weakness and malnutrition before the onset of SICH. Finally, long-term intensive glycemic control may be about low or extremely low HbA1c after SICH. Regular health monitoring should be carried out for people with low or very low HbA1c levels, and the causes of abnormal HbA1c levels should be identified. HbA1c detection at least twice a year is recommended for patients with high risk of ICH, which may benefit them with early intervention. Early intervention refers to the following: (1) It is needed to improve the liver function examination early, being alert for potential liver dysfunction. On the basis of other studies, it has been shown that there is a lag in the increase of liver enzymes after liver function injury. Albumin dysfunction can be adopted as a new potential indicator of liver function injury (39). (2) We should look for malabsorption problems such as eating disorders or celiac disease to improve the nutritional status. (3) Being alert for severe hypoglycemia, we attempt to use appropriate antidiabetic drugs for individualized treatment. Previous randomized controlled trials revealed that intensive glycemic control does not reduce the cardiovascular risk in patients with diabetes (40–42). Meanwhile, strict glycemic control increased the risk of severe hypoglycemia, and the incidence of severe hypoglycemia events related to intensive treatment was two to three times higher than that of the non-intensive treatment group (43–45). From this point of view, long-term blood glucose should not be overcontrolled in diabetic patients with a high risk of SICH (46). Likewise, routine monitoring and routine HbA1c detection at admission are of great significance for patients with SICH to possibly prevent severe damage. At 3 months, the blood glucose threshold of symptoms and neuroendocrine response returned to normal with the recovery of glucagon response (47). For SICH patients with a low HbA1c level, it may be necessary to ensure enough brain energy and actively give non-surgical interventions including hypertonic drugs, antihypertensive drugs, and surgical interventions such as craniotomy or minimally invasive surgery (MIS).

HbA1c is a biomarker used to monitor blood glucose, which can evaluate the average blood glucose status within 2 to 3 months. Compared with other diabetes detection (such as fasting blood glucose or glucose tolerance test), HbA1c measurement does not need fasting, has high stability, is less affected by acute physiological disorders, and is more convenient for clinical application (23, 24). Some studies have shown that HbA1c is related to poor prognosis in patients with SICH (48, 49). These results are consistent with earlier studies indicating that patients with newly diagnosed diabetes based on the HbA1c criteria have a poor long-term prognosis after acute cerebral hemorrhage (50). Small cohort studies suggested that HbA1c is a better predictor of adverse outcomes in patients with SICH (25–27). Besides, HbA1c can better predict the symptomatic hemorrhagic transformation of acute ischemic stroke after thrombolysis (26, 51). These findings support the results of a large cohort study showing a J-type relationship between HbA1c and the risk of SICH. The lowest risk observed in HbA1c was 6.5% (52). Taken together, HbA1c levels might affect the incidence and prognosis of an acute cerebral hemorrhage, which is consistent with our findings. However, our outcome is contrary to a previous study showing that HbA1c was not associated with clinical outcome in patients with SICH (28).

At the same time, our study showed that when HbA1c was 5.60–6.10%, compared with patients without DM, DM patients with reasonable pre-stroke glycemic control had a lower mortality rate after SICH, but there was no statistical difference (1.00 vs. 1.84%, *p* = 0.543). This positive relationship could be linked to speculations that basement membrane thickening and endothelial cell proliferation caused by long-term diabetes before stroke reduce the risk of rebleeding after cerebral vascular rupture. In diabetic patients, increased levels of coagulation and plasminogen activator inhibitors are associated with the decreased fibrinolytic activity, as well as reduced bleeding volume and risk of rebleeding (53). However, this evidence contradicts previous studies that diabetes is a predictor of poor outcome after SICH (7, 54, 55).

There are several limitations in our current research. First of all, there is no follow-up data in this study, so long-term prognosis data are not available. At the same time, there were no imaging data and blood samples. The volume and location of the hematoma were not recorded. Secondly, in this study, patients without or with HbA1c measurement presented major baseline differences (Table 1). At the same time, the correlation between HbA1c and mortality was not statistically significant in the sensitivity analysis among patients with GCS and NIHSS scores (full details are given in Appendices 1, 2). The reason may be that patients with missing values have more severe neurological deficits and higher mortality, leading to possible selection bias.

## Conclusion

In summary, this study mainly discusses the effect of low HbA1c on the prognosis of patients with SICH and expounds the possible mechanism. This study demonstrates that low or extremely low HbA1c is proportionally related to higher short-term mortality after SICH, regardless of whether patients have DM or not. Low or extremely low HbA1c may be associated with liver disease, long-term malnutrition, and excessive glycemic control in diabetes. It is very important for clinical decision-making to find the cause of low or extremely low HbA1c. These findings and their clinical significance need to be evaluated by future randomized clinical trials.

## Data Availability Statement

The datasets generated for this study are available on request to the corresponding author.

## Ethics Statement

Ethical review and approval was not required for the study on human participants in accordance with the local legislation and institutional requirements. Written informed consent for participation was not required for this study in accordance with the national legislation and the institutional requirements.

## Author Contributions

PL and LC: study design, analysis and interpretation, and primary responsibility for writing the manuscript. YW, KK, HG, ZL, LL, YW, and XZ: study design, data interpretation, critical revision of the manuscript for important intellectual content, and supervision of the study. HG: data statistics. All authors: contributed to the article and approved the submitted version.

## Conflict of Interest

The authors declare that the research was conducted in the absence of any commercial or financial relationships that could be construed as a potential conflict of interest.
