# Supplementary material for: Relationship Between Glycosylated Hemoglobin and Short-Term Mortality of Spontaneous Intracerebral Hemorrhage
Source: Front Neurol. 2021 Apr 16;12:648907. doi: 10.3389/fneur.2021.648907 (PMC8085396; doi:10.3389/fneur.2021.648907)
Supplement: Supplementary file 1 [file Data_Sheet_1.docx]

**Appendix 1 sensitivity analysis of patients with recorded GCS and NIHSS**

| **HbA1c** | **N** | **Death,n（%）** | **Univariate analysis** | | **Multivariate analysis*** | |
| --- | --- | --- | --- | --- | --- | --- |
|  |  |  | **P Value** | **OR(95%CI)** | **P Value** | **aOR(95%CI)** |
| ≤ 5.10% | 2025 | 36(1.78) | - | - | - | - |
| 5.10-5.60% | 2140 | 46(2.15) | 0.389 | 1.21(0.78-1.89) | 0.084 | 1.5(0.95-2.38) |
| 5.60-6.10% | 1725 | 32(1.86) | 0.86 | 1.04(0.65-1.69) | 0.424 | 1.23(0.74-2.04) |
| ≥ 6.10% | 1695 | 53(3.13) | 0.008 | 1.78(1.16-2.74) | 0.069 | 1.57(0.97-2.56) |

The multivariate model was adjusted for age, sex, BMI, diastolic blood pressure, atrial fibrillation, myocardial infarction, hypertension history, diabetes history, lipid metabolism disorder, peripheral vascular disease, antiplatelet drugs, antihypertensive drugs, hypoglycemic drugs, smoking history, drinking history, hypoglycemic therapy after the onset of the disease. GCS and NIHSS are included.

**Appendix 2 sensitivity analysis: Stratified analysis of association between HbA1c levels and short-term mortality after admission in patients of SICH with or without DM.**

| **HbA1c** | **DM** | | | | | | **non-DM** | | | | | | |
| --- | --- | --- | --- | --- | --- | --- | --- | --- | --- | --- | --- | --- | --- |
|  |  |  |  |  |  |  |  | | | | | |  |
|  | **N** | **Death,n（%）** | **Univariate analysis** | | **Multivariate analysis*** | | **N** | **Death,n（%）** | **Univariate analysis** | | **Multivariate analysis*** | | |
|  |  |  | **P Value** | **OR(95%CI)** | **P Value** | **aOR(95%CI)** |  |  | **P Value** | **OR(95%CI)** | **P Value** | **aOR(95%CI)** | |
| ≤ 5.10% | 44 | 1(2.27) | - | - | - | - | 1981 | 35(1.77) | - | - | - | - | |
| 5.10-5.60% | 39 | 1(2.56) | 0.931 | 1.13(0.07-18.72) | 0.677 | 1.96(0.08-46.20) | 2101 | 45(2.14) | 0.389 | 1.22(0.78-1.90) | 0.092 | 1.5(0.94-2.39) | |
| 5.60-6.10% | 71 | 0(0.00) | 0.963 | (<0.01->999.99) | 0.932 | (<0.01->999.99) | 1654 | 32(1.93) | 0.708 | 1.1(0.68-1.78) | 0.364 | 1.27(0.76-2.13) | |
| ≥ 6.10% | 495 | 22(4.44) | 0.503 | 2.00(0.26- 15.20) | 0.657 | 1.70(0.17-17.49) | 1200 | 31(2.58) | 0.12 | 1.48(0.91-2.40) | 0.203 | 1.4(0.83-2.36) | |

The multivariate model was adjusted for age, male, BMI, diastolic blood pressure, atrial fibrillation, myocardial infarction, hypertension history, diabetes history, lipid metabolism disorder, peripheral vascular disease, antiplatelet drugs, antihypertensive drugs, hypoglycemic drugs, smoking history, drinking history, hypoglycemic therapy after the onset of the disease. GCS and NIHSS are included.
